# Supplementary material for: An Extreme Value Theory Model of Cross-Modal Sensory Information Integration in Modulation of Vertebrate Visual System Functions
Source: Front Comput Neurosci. 2019 Feb 26;13:3. doi: 10.3389/fncom.2019.00003 (PMC6400236; doi:10.3389/fncom.2019.00003)
Supplement: Supplementary file 1 [file Data_Sheet_1.PDF]

Supplementary Material:
An Extreme Value Theory Model of Cross-Modal Sensory Information Integration in Modulation of Vertebrate Visual System Functions

1 GENERALIZED PARETO DISTRIBUTION FITTING

We used a Matlab implementation of the Bayesian Information Criterion (BIC) method [1]. As our data is continuous (RGC responses are positive real numbers), the list of continuous distributions the BIC implementation tries to fit are: Beta, Birnbaum-Saunders, Exponential, Extreme Value, Gamma, Generalized Extreme Value, Generalized Pareto, Inverse Gaussian, Logistic, Log-logistic, Log-Normal, Nakagami, Normal, Rayleigh, Rician, t Location-Scale, and Weibull.

The selection of the Generalized Pareto distribution was based on the highest probability score output by the BIC method, considering all of the above distributions as possible candidates. Included are plots for the CDFs of the top-4 fitting distributions that the BIC function returns against our data, sorted in order from best to worst (in the legend). The "empirical" curves in the plots represent the the raw data (RGC responses without olfactory stimulation) collected from the wet-bench experiments.

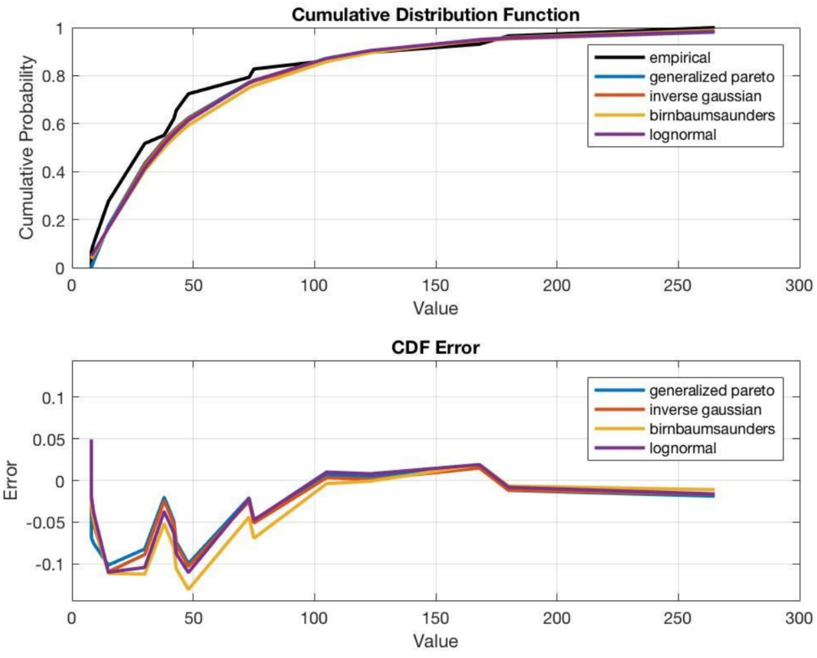

Figure S1. Experiment 1. Cumulative Distribution Functions and its corresponding error plots for the top - 4 distributions returned by BIC method. The "empirical" curves in the plots represent the the raw data (RGC responses without olfactory stimulation).

[1] https://github.com/dcherian/tools/blob/master/misc/allfitdist.m
